# Analyzing the correlation between low proportion of hobnail features in papillary thyroid carcinoma and clinical aggressiveness risk

**DOI:** 10.1007/s12020-024-03854-9

**Published:** 2024-07-06

**Authors:** Wen-Shun Liu, Yan-Ting Duan, Guo-Qing Ru, Wan-Yuan Chen, Yuan Chen, Tian Lv, Ju-Yong Liang, Guo-Wan Zheng, Jia-Jie Xu

**Affiliations:** 1 Jinzhou Medical University, Jinzhou, 121001 Liaoning China; 2 Otolaryngology & Head and Neck Center, Cancer Center, Department of Head and Neck Surgery, Zhejiang Provincial People’s Hospital, 310014 Hangzhou, Zhejiang China; 3 Key Laboratory of Endocrine Gland Diseases of Zhejiang Province, 310014 Hangzhou, Zhejiang China; 4 Clinical Research Center for Cancer of Zhejiang Province, 310014 Hangzhou, Zhejiang China; 5 Cancer Center, Department of Pathology, Zhejiang Provincial People’s Hospital (Affiliated People’s Hospital), Hangzhou Medical College, Hangzhou, Zhejiang China

**Keywords:** Hobnail features, Low proportion of hobnail features (<30%), Papillary Thyroid Carcinoma, Thyroid Tumors

## Abstract

**Purpose:**

Hobnail features may enhance the clinical aggressiveness of papillary thyroid carcinoma (PTC). However, whether a low proportion (<30%) of these features contributes to increased PTC aggressiveness remains unclear. This study investigated whether PTC cases with a low proportion hobnail features (<30%) exhibit clinical invasiveness and pathological features of aggressiveness.

**Methods:**

Pathological specimens from patients with postoperatively diagnosed PTC were retrospectively analyzed. Among them, 29 PTC cases with a low proportion of hobnail features (<30%) were compared with 173 consecutive classical PTC (cPTC) cases. Data regarding age at presentation, sex, tumor size, number of tumors, and histological characteristics were obtained by reviewing electronic medical records. Postoperative information was obtained during follow-up visits and telephone interviews.

**Results:**

Twenty-nine patients with PTC with a low proportion of hobnail features (<30%) were identified, exhibiting a median age of 34 years. At a median follow-up of 31 (IQR, 23–37) months, two patients had recurrent disease in the PTC with a low proportion of hobnail features (<30%) group, whereas there was no recurrence in the cPTC group. No distant metastasis and postoperative mortality were observed in either group. Compared with the cPTC group, patients with PTC and a low proportion of hobnail features exhibited larger tumor volumes and higher susceptibility to capsular invasion and lymph node metastasis. Tumor size and hobnail features emerged as independent risk factors for lymph node metastasis.

**Conclusion:**

PTC with a low proportion hobnail features (<30%) and larger tumor volumes are associated with the occurrence of lymph node metastasis. A low proportion of hobnail features (<30%) in PTC may heighten invasiveness, elevating the risk of recurrence.

## Introduction

Thyroid cancer is the most prevalent type of endocrine malignancy, primarily appearing in a well-differentiated form. Notably, papillary thyroid carcinoma (PTC) is the most common histological type, constituting approximately 85% of thyroid cancers [1]. Most patients with PTC have a favorable prognosis after surgical treatment, with a 10-year survival rate of up to 90% [2]. However, some PTC cases exhibit high invasiveness, leading to lymph node metastasis, recurrence, and resistance to radioiodine therapy [3]. Therefore, it is critical to identify patients at higher risk of recurrence and conduct more extensive surgery or focused postoperative follow-up. Traditionally, histological features have been used to assess the clinical malignancy of PTC. Recent findings highlight that different histological subtypes present in PTC can influence tumor aggressiveness, such as the presence of tall and columnar cells [4]. Hobnail features are aggressive histological patterns that appear in PTC, characterized by the presence of hobnail cells within the tumor tissue, displaying a growth pattern of micropapillary and an increased nuclear-cytoplasmic ratio in tumor cells [5]. The World Health Organization (WHO) classifies PTC into multiple subtypes, among which the hobnail subtype is considered a rare and highly invasive subtype, constituting 1.08% of all PTC cases [6]. The hobnail subtype of papillary thyroid carcinoma (HPTC) is rare and exhibits an aggressive clinical course, often leading to frequent distant metastases and the occurrence of iodine resistance [7]. A significant proportion of patients succumb to the disease. Currently, tumors with hobnail features exceeding 30% are classified as HPTC [8]. Compared with those with classical papillary thyroid carcinoma (cPTC), patients diagnosed with HPTC have a worse prognosis. Numerous studies suggest that a high proportion of hobnail features intensifies PTC aggressiveness [9, 10]. However, it remains unclear whether a low proportion of these features (<30%) similarly impacts PTC aggressiveness. Limited research has been conducted on the disparities between PTC exhibiting hobnail features < 30% and cPTC. This study retrospectively analyzed the clinical and pathological data of 29 cases of PTC with a low proportion of hobnail features (<30%) treated at our hospital and compared them with 173 cases of cPTC treated during the same period. This study aimed to explore the clinical characteristics of patients with PTC having a low proportion of hobnail features (<30%) and analyze the impact of these features on PTC invasiveness. Our findings are anticipated to shed light on the understudied realm of PTC with a low proportion of hobnail features (<30%). By delving into the clinical characteristics and potential impacts on PTC invasiveness, this study strives to augment our understanding of this specific PTC subtype.

## Materials and methods

We conducted a retrospective analysis of patients diagnosed with primary PTC who underwent initial surgical treatment at our hospital between 2017 and 2021. Two senior pathologists determined the proportion of these features and independently determined the percentage of hobnail areas in PTC tumor pathology slides to assess the presence of hobnail features in PTC tumor pathology slides. The average value was used as the final percentage value for hobnail features unless a disagreement of 3% or more was present. In these cases, slides were reviewed until the results are consistent, setting 5% and 25% (<30%) as the minimum and maximum cutoff. In total, 173 cPTCs and 29 PTCs with a low proportion of hobnail features (<30%, as hobnail features group in our reserch) were identified and included in this study. Clinicopathological information for each patient, including age, sex, tumor multifocality, tumor size, capsular invasion, and occurrence of multifocal lymph node metastasis (defined as central region and lateral neck lymph node metastases), was obtained from the patient database. Tumor staging followed the tumor–node–metastasis classification as per the 8th edition of the American Joint Committee on Cancer staging system for thyroid carcinoma. Follow-up data were obtained via telephone and postoperative examinations. This study was approved by the Ethics Committee of our Hospital (Approval No.: Zhe Ren Yi Lun Shen 2023 Other [206]).

### Mutation analysis

Genomic DNA was extracted from frozen tissues after surgery using the QIAamp DNA FFPE Tissue Kit (Item number: 56404, Qiagen, Italy), according to the manufacturer’s protocol. Mutational analysis was performed for BRAF (NM_004333.4) using the Hunam BRAF Gene Mutation Qualitative Detection Kit (Fluorescent PCR) (Item number: TB004, Beijing ACCB Biotech Ltd).

### Statistical analysis

Data were analyzed using IBM SPSS version 26.0 (IBM Corp., Armonk, NY, USA). Normally distributed variables were expressed as means and standard deviations, and differences were analyzed using t-tests. Nonnormally distributed variables were represented as medians and interquartile ranges (IQRs) and were analyzed using the rank-sum test. Categorical variables were subjected to statistical analysis using the chi-square and Fisher’s exact tests. In this study, a *P*-value < 0.05 was considered statistically significant, and logistic regression models were employed to analyze relevant data.

## Results

### Clinicopathologic characteristics of PTC with hobnail features

Between 2017 and 2021, our study included 202 cases of PTC, all categorized as cPTC. Among these cases, 29 exhibited a low proportion of hobnail features (<30%, ranging between 5% and 25%). The median age was 34 (27–50) years, and the median tumor size was 15.0 (9.5–25.0) mm. Tumor staging in the hobnail features group revealed 19 cases (65.6%) classified as T1, 9 (31.0%) as T2, and 1 (3.4%) as T3. Analysis of the invasion level of hobnail features group revealed that 11 (37.9%) had capsular invasion, and 8 (27.6%) presented with multifocality. At the initial diagnosis, 24 cases (82.8%) showed lymph node metastasis, including 12 (41.4%) classified as N1b (Table 1).

**Table 1:** Clinicopathological characteristics of PTC with hobnail features and cPTC

		Total (*n* = 202)	PTC with hobnail features (*n* = 29)	cPTC (*n* = 173)	*P*-value
Age [years, *M*(IQR)]			34 (27–50)	42 (35–51)	0.022
BMI [kg/m^2^, *M*(IQR)]			23.14 (20.55–26.50)	23.40 (20.80–25.40)	0.903
TSH [mlU/L, *M*(IQR)]			2.19 (1.33–3.52)	1.72 (1.10–2.38)	0.098
Size [mm, *M*(IQR)]			15.0 (9.5–25.0)	6.00 (4.00–9.50)	0.0001
Gender					0.038
	Male	58 (28.7%)	13 (44.8%)	45 (26.0%)	
	Female	144 (71.3%)	16 (55.2%)	128 (74.0%)	
Bilateral tumors					0.025
	Yes	39 (19.3%)	10 (34.5%)	29 (16.8%)	
	No	163 (80.7%)	19 (65.5%)	144 (83.2%)	
Multifocality					0.754
	Yes	51 (25.2%)	8 (27.6%)	43 (24.9%)	
	No	151 (74.8%)	21 (72.4%)	130 (75.1%)	
Lymph node metastasis					0.0001
	Yes	83 (41.1%)	24 (82.8%)	59 (34.1%)	
	No	119 (58.9%)	5 (17.2%)	114 (65.9%)	
Multiple lymph node metastasis					0.0001
	Yes	58 (28.7%)	19 (65.5%)	39 (22.5%)	
	No	144 (71.3%)	10 (34.5%)	134 (77.5%)	
Capsular invasion					0.023
	Yes	44 (21.8%)	11 (37.9%)	33 (19.1%)	
	No	158 (78.2%)	18 (62.1%)	140 (80.9%)	
Recurrence					0.02
	Yes	2 (1.0%)	2 (6.9%)	0 (0%)	
	No	200 (99.0%)	27 (93.1%)	173 (100%)	
T					0.0001
	T1	187 (92.6%)	19 (65.6%)	168 (97.1%)	
	T2	14 (6.9%)	9 (31.0%)	5 (2.9%)	
	T3-4	1 (0.5%)	1 (3.4%)	0 (0.0%)	
N					0.046
	N1a	55 (27.2%)	12 (41.4%)	43 (24.9%)	
	N1b	28 (13.9%)	12 (41.4%)	16 (9.2%)	
Stage					0.004
	I	193 (95.5%)	24 (82.8%)	169 (97.7%)	
	II	8 (4.0%)	4 (13.8%)	4 (2.3%)	
	III	1 (0.5%)	1 (3.4%)	0 (0%)	
BRAF mutation					0.381
	Yes		6 (20.6%)	35 (20.2%)	
	No		5 (17.2%)	51 (29.4%)	

*BMI* Body Mass Index, *TSH* Thyroid stimulating hormone, *PTC* Papillary thyroid carcinoma

### Treatment and prognosis

All patients underwent total thyroidectomy or lobectomy with lymph node dissection. The median follow-up period spanned 31 (IQR, 23–37) months. Follow-up data revealed that 2 patients (6.9%) experienced recurrence (defined as the discovery of contralateral thyroid or cervical lymph node metastasis during postoperative review, confirming cancer recurrence) in hobnail features group. In contrast, no instances of recurrence were observed in the cPTC group. Additionally, none of the patient in hobnail features group experienced distant metastases, and no deaths were reported in either group (Table 1).

### Comparison between PTC with hobnail features and cPTC

Statistical analysis of the enrolled patients revealed a higher proportion of female patients in both groups. The male-to-female ratio was 1:1.2 in the hobnail features group, whereas in the cPTC group, it was 1:2.84. The median age at diagnosis for the hobnail features and cPTC groups were 34 (27–50) and 42 (35–51) years, respectively Table 2. The median tumor diameter was 15 (9.5–25.0) mm in the hobnail features group and 6 (4.0–9.5) mm in the cPTC group, indicating a significant difference between the two groups (*P* = 0.0001). Subsequent comparisons revealed no significant difference in the occurrence of multifocality between the two groups. However, the proportion of patients with lymph node metastasis was higher in the hobnail features group than in the cPTC group (*P* = 0.0001) (Table 1). Tumor size (odds ratio [OR]: 1.088, *P* = 0.011) and hobnail features (OR: 4.519, *P* = 0.009) were identified as independent risk factors for lymph node metastasis (Table 3). Compared with the cPTC group, the ultrasound images in the hobnail features group more frequently presented irregular or lobulated margins (*P* = 0.023), microcalcifications (*P* = 0.036), and internal vascularity (*P* = 0.001) (Table 4).

**Table 2:** Univariate logistic regression analysis of variables predicting lymph node metastasis in PTC

Variables	LNM (*n* = 83)	NLNM (*n* = 119)	OR (95%CI)	*P*-value
Age			0.952 (0.927–0.977)	0.001
Size			1.154 (1.088–1.224)	0.001
BMI			1.032 (0.967–1.101)	0.35
Bilateral tumors			2.164 (1.066–4.392)	0.033
No	61	102		
Yes	22	17		
Capsular invasion			3.723 (1.840–7.534)	0.001
No	54	104		
Yes	29	15		
Pathologic characteristics			9.275 (3.366–25.553)	0.001
PTC with hobnail features	24	5		
cPTC	59	114		

*LNM* Lymph node metastasis, *NLNM* No lymph node metastasis, *TSH* Thyroid stimulating hormone, *PTC* Papillary thyroid carcinoma, *cPTC* Classical papillary thyroid carcinoma

**Table 3:** Multivariate logistic regression analysis of variables predicting lymph node metastasis in PTC

Variables	LNM (*n* = 83)	NLNM (*n* = 119)	OR (95%CI)	*P*-value
Age			0.960 (0.932–0.988)	0.005
Size			1.088 (1.020–1.161)	0.011
Bilateral tumors			1.398 (0.594–3.288)	0.443
No	61	102		
Yes	22	17		
Capsular invasion			2.540 (1.136–5.677)	0.023
No	54	104		
Yes	29	15		
Pathologic characteristics			4.519 (1.449–14.093)	0.009
PTC with hobnail features	24	5		
cPTC	59	114		

*LNM* Lymph node metastasis, *NLNM* No lymph node metastasis, *PTC* Papillary thyroid carcinoma, *cPTC* Classical papillary thyroid carcinoma

**Table 4:** US characteristics of PTC with hobnail features and cPTC

		Total (*n* = 202)	PTC with hobnail features (*n* = 29)	cPTC (*n* = 173)	*P*-value
Composition					*1*
	Solid		29	171	
	Solid and cystic		0	2	
Margin					0.023
	Regular	109 (54.0%)	10 (34.5%)	99 (57.2%)	
	Irregular/lobulated	93 (46.0%)	19 (65.5%)	74 (42.8%)	
Taller than wide					0.791
	No	86 (42.6%)	13 (44.8%)	73 (42.2%)	
	Yes	116 (57.4%)	16 (55.2%)	100 (57.8%)	
Macrocalcifications					0.036
	No	92 (45.5%)	8 (27.6%)	84 (48.6%)	
	Yes	110 (54.5%)	21 (72.4%)	89 (51.4%)	
Internal vascularity					0.001
	No	137 (67.8%)	12 (41.4%)	125 (72.3%)	
	Yes	65 (32.2%)	17 (58.6%)	48 (27.7%)	
Lymph node					0.322
	No	128 (63.4%)	16 (55.2%)	112 (64.7%)	
	Yes	74 (36.6%)	13 (44.8%)	61 (35.3%)	

*US* Ultrasound, *PTC* Papillary thyroid carcinoma, *cPTC* Classical papillary thyroid carcinoma

### Pathological features and BRAF mutation

**Fig. 1:**
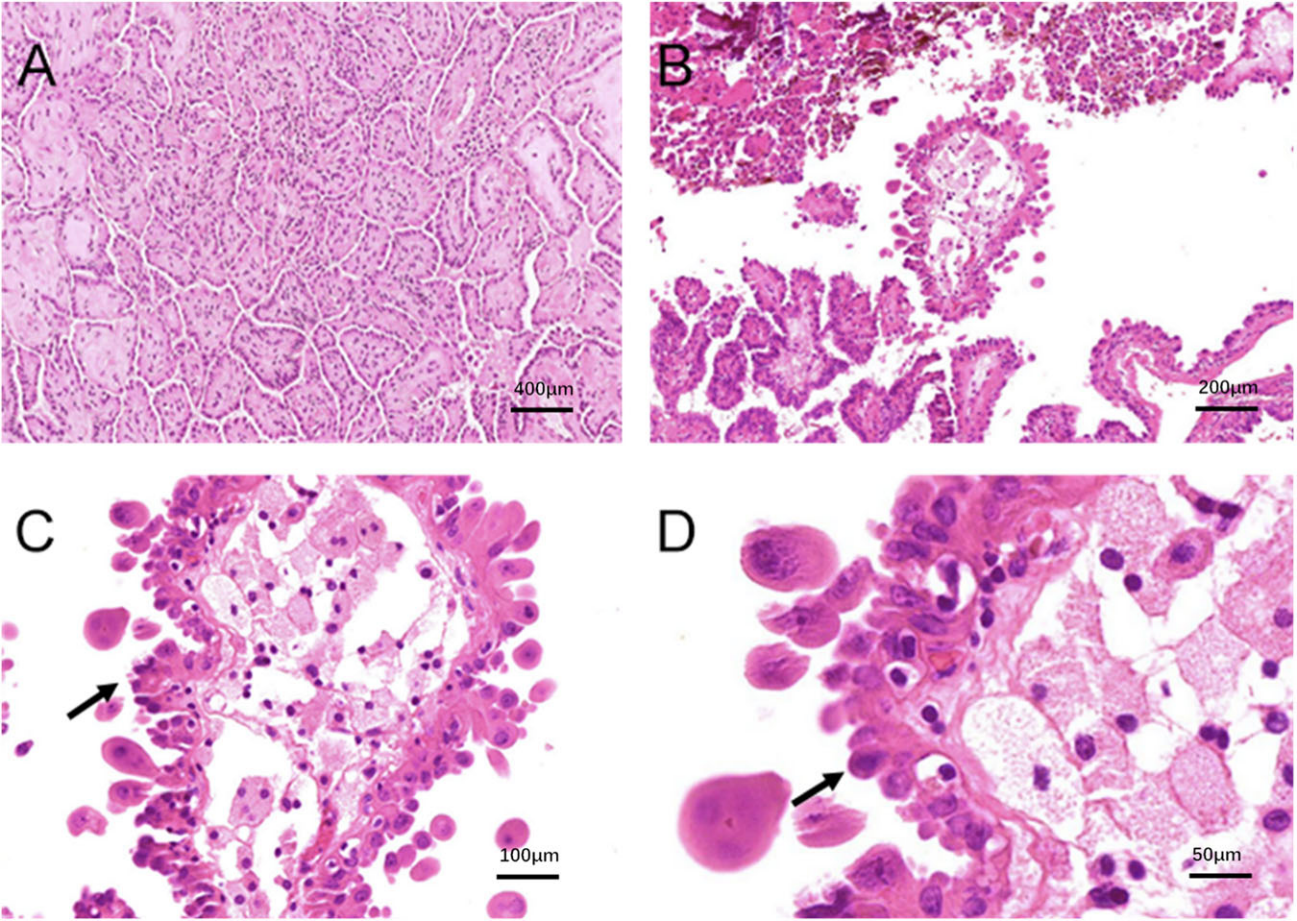
Histological features of papillary thyroid carcinoma and hobnail features in a section. **A** classical papillary thyroid carcinoma (H&E ×4); **B** A micropapillary fragment showing Hobnail feathers (H&E ×10); **C** loss of cellular polarity, increased eosinophilic cytoplasm, increased nuclear to cytoplasmic ratios, and pleomorphic nuclei mostly located in the apex of the cytoplasm that gives the characteristic Hobnail features (H&E ×20); **D** The detail of Hobnail features (black arrow) (H&E ×40). H&E hematoxylin and eosin staining

In a retrospective analysis of pathological slides from hobnail features group in our study, microscopic observations revealed that the tumors exhibited common histological features of PTC. All tumors showed the nuclear features of cPTC. Additionally, scattered tumor cells demonstrated a loss of polarity or adhesion arranged in papillary or micropapillary patterns within the tumor tissue. These tumor cells varied in size, had abundant eosinophilic cytoplasm, and were acidophilic. The nuclei were located at the apex of the cells, producing projections at the corresponding surface positions and demonstrating marked micropapillary morphological changes (Fig. 1). A BRAF mutation was found in 6/11 (54%) patients in the hobnail features group (<30%) and in 35/86 (40.7%) in the cPTC group. There was no significant difference between the two groups (Table 1).

## Discussion

Our study explored the association between tumor invasiveness and the risk of tumor development in patients with PTC with a low proportion (<30%) in clinical practice. Amacher et al. [10] identified hobnail features (representing an aggressive histological characteristic) in poorly differentiated thyroid carcinoma (PDTC) and anaplastic thyroid carcinoma (ATC), noting a higher likelihood of these features in PDTC. WHO formally designated PTC with hobnail features comprising >30% of the tumor as HPTC in 2017 [6]. The histological morphology of HPTC is characterized by infiltrative complex micropapillary structures, tumor cells exhibiting poor adhesion and losing the typical polar arrangement of normal thyroid tissue. This alteration is specifically manifested by the relocation of tumor cell nuclei to the apical surface, presenting an overall hobnail morphology [11–14]. HPTC has been a focal point in thyroid cancer research, with clinical data indicating worse prognosis and higher levels of recurrence and tumor-related mortality compared to cPTC [15, 16]. Previous studies on PTC with a low proportion hobnail features (<30%) have shown that these are still aggressive and are not significantly different from HPTC [14]. Therefore, PTC with a low proportion of hobnail features (<30%) should be taken seriously to improve the patient’s tumor control level. Currently, there are fewer studies on patients with PTC with a low proportion hobnail features (<30%), and it remains unclear whether this group of patients should be administered standardized treatment as in the case of highly aggressive tumors.

We conducted a retrospective study on 29 cases diagnosed as PTC with a low proportion of hobnail features (<30%) from 2017 to 2021. Previous studies have shown that the prevalence of HPTC is 1.08% [8], the prevalence of PTC patients with a low proportion of hobnail features (<30%) in our cohort was 0.8%, which is consistent with previous studies and illustrates the rarity of this histological feature. We analyzed the histological, molecular, and ultrasonic characteristics of our samples. The results revealed that the hobnail features group significantly differed from the cPTC group in the aggressive characteristics. Additionally, a low proportion of hobnail features was associated with known adverse prognostic factors, including larger tumors at diagnosis, lymph node metastasis, and recurrence. There have been reports showing that the prevalence of thyroid cancer in women is about three times that in men, HPTC in men ranges from 35% to 38.2% [8, 17], whereas that of PTC with a low proportion of hobnail features (<30%) in men ranges from 43.1% to 44.4% [17, 18]. In our cohort, the prevalence was 44.8% in men and 55.2% in women, which is consistent with the results of previous studies. Both young and older adult patients have hobnail features, and the proportion of hobnail features did not correlate with age. [19]. Recent data indicate that the average age of patients with HPTC range from 41.8 to 52.3 years [8, 12, 19]. For the patients with PTC with a low proportion of hobnail features (<30%), the mean age is 46.3 years [17]. Conversely, the median age for patients with hobnail features group was 34 years, and for patients with cPTC group was 42 years in our cohort (Table 1). Compared with previous data, our results indicate that the hobnail features group presented at a younger age, with a median age lower than that of the cPTC group. Consistent with cancer incidence statistics, the onset age for thyroid cancer tends to be younger [20], which may relate to our findings. Thus, attention should be directed towards younger patients with more rapidly progressing tumors. Additionally, tumor size has been identified as a risk factor for lymph node metastasis, recurrence, and mortality [21], with the median size of PTC exhibiting hobnail features (<30%) being 18 mm, whereas the mean size reported in prior studies is 28 mm [17, 18]. In our cohort, the median tumor size in the hobnail features group was 15 mm, this is consistent with previous research. Previous studies have demonstrated that the probability of bilateral tumors in PTC with a low proportion (<30%) of hobnail features is 23.4% [17]. Our results indicate that the probability in the hobnail features group is 34.5%, whereas in the cPTC group, it is 16.8% (*P* = 0.025). Additionally, earlier research found that the probability of lymph node metastasis in PTC with a low proportion (<30%) of hobnail features is 50% [8]. Our findings reveal that the probability of lymph node metastasis in the hobnail features group is 82.8%, compared with 34.1% in the cPTC group (*P* = 0.0001). The probability of capsular invasion of the hobnail features group is 37. 9%, whereas that of the cPTC group is 19. 1% (*P* = 0.023). This data shows an increased probability of capsular invasion in the hobnail features group, which may also contribute to increased cancer cell invasion capability and tumor development. Additionally, when tumor cells invade the thyroid capsule, they increase the risk of tumor lymph node metastasis [22]. Multivariate logistic regression analysis indicated a correlation between hobnail features, tumor diameter, and occurrence of lymph node metastasis (Table 3), this suggests that a low proportion of hobnail features (<30%) increases the risk of lymph node metastasis. BRAF mutations are associated with a higher risk of death [23]. We also analyzed BRAF mutations in the two patient groups. In our cohort, the BRAF mutation rate was 54% in the hobnail features group and 40.7% in the cPTC group; the difference was not significant. Other studies have shown that the recurrence rate for PTC with a low proportion of hobnail features (<30%) is 8.7% and the distant metastasis rate ranges from 0 to 14% [8, 17]. We followed all patients for a median of 31 months (IQR, 23–37). The recurrence rate in the hobnail features group was 6.9% (2 patients); no relapses occurred in the cPTC group, and no patients developed distant metastasis in either group in our cohort. By comparing the histological and molecular characteristics of the two groups of patients, we found that the hobnail features group had a higher proportion of larger tumor tissue, higher lymph node metastasis, and recurrence rates, compared with the cPTC group. Our study results further underscore that the presence of a low proportion of hobnail features (<30%) increases the invasiveness of PTC.

## Conclusions

We analyzed and statistically evaluated the histological features and patient prognosis data in cases of PTC with a low proportion of hobnail features (<30%). Our results indicate that even when the proportion of hobnail features is lower (<30%), it can contribute to increased invasiveness of PTC. Further comprehensive research is essential to explore and elucidate the reasons and mechanisms underlying the appearance of a low proportion of hobnail features (<30%) in PTC. This exploration will provide a theoretical basis for better understanding the relationship between this feature and PTC invasiveness, serving as a reference to optimize treatment strategies for patients with PTC exhibiting a low proportion (<30%) of hobnail features in clinical practice. There are some limitations to this study. First, the sample size was limited, especially for multivariate analyses. Second, because the follow-up period of many of the patients was short, we were unable to assess their survival. Therefore, studies with larger samples and long follow-ups should be performed to determine long-term prognosis.
